# Cancer screening discrepancies among Black people in Canada: a scoping review

**DOI:** 10.1186/s12889-026-27317-0

**Published:** 2026-04-22

**Authors:** Aloysius Nwabugo Maduforo, Prosper Komolafe, Tiphanie Okorie, Caitlin McClurg, Yinka Oladele, Bukola Salami

**Affiliations:** 1 Department of Community Health Sciences, Cumming School of Medicine, University of Calgary, Calgary, AB Canada; 2 Libraries and Cultural Resources, University of Calgary, Calgary, AB Canada; 3 The Oladele Foundation - African Cancer Support Group, Calgary, AB Canada

**Keywords:** Cancer screening, Black people in Canada, Health disparities, Socioeconomic barriers, Health Access

## Abstract

**Background:**

Cancer is a major cause of death in Canada, and Black people in Canada experience notable disparities in screening. This scoping review summarises existing evidence on cancer screening among Black people in Canada and identifies key barriers, facilitators, and implications for policy and practice.

**Methods:**

The review followed Arksey and O’Malley’s five-stage scoping review framework and adhered to the PRISMA-ScR guidelines. We conducted a systematic search of Sociological Abstracts, SocINDEX, PsychINFO, Embase, Social Science Citation Index, Social Work Abstracts, CINAHL, Scopus, Gender Studies Database, PubMed, ProQuest, and Web of Science. Articles were included if they focused on cancer screening among Black people in Canada, excluding literature reviews and grey literature. Data extraction covered study characteristics, screening methods, rates, and identified barriers and facilitators.

**Results:**

Nineteen studies met the inclusion criteria, with a geographical concentration in Ontario (11 studies), followed by Nova Scotia (3), Alberta (2), and Quebec (1). The studies predominantly addressed cervical (58%) and breast cancer (26%), utilizing cross-sectional and retrospective cohort designs. Key barriers to screening included socioeconomic constraints, cultural stigmas, and structural racism within the healthcare system. Recommended interventions included culturally sensitive education, patient navigation services, and advanced screening technologies.

**Conclusions:**

Significant disparities in cancer screening rates persist among Black people in Canada due to systemic, cultural, and socioeconomic barriers. Tailored interventions and equitable policies are needed to improve access and participation in screening services. Future research should prioritise longitudinal studies and incorporate comprehensive socioeconomic and cultural data to better inform strategies that support early detection and reduce cancer-related mortality.

**Supplementary Information:**

The online version contains supplementary material available at 10.1186/s12889-026-27317-0.

## Introduction

Cancer remains one of the leading causes of mortality in Canada and globally, accounting for approximately one in six deaths worldwide. In Canada, it is estimated that about two in five people will be diagnosed with cancer at some point during their lifetime [1–3]. Although advances in early detection and treatment have improved overall cancer outcomes, these gains have not been equitably distributed across all population groups. In Canada, growing evidence indicates that Black people experience disproportionate cancer burdens, including higher mortality from breast, colorectal, prostate, and pancreatic cancers compared with non-Black populations, despite universal healthcare coverage [4]. Inequities in screening are an important contributor to these disparities.

Black communities, including many immigrant populations, are consistently underrepresented in breast, cervical, and colorectal screening programs, which places them at greater risk of late-stage diagnosis and poor outcomes [4]. Sub-Saharan African immigrant women are significantly more likely to be under-screened for cervical cancer, with an adjusted prevalence ratio of 3.6 (95% confidence interval 2.4 to 5.4) for never having had a Pap test compared to Canadian-born women [5, 6]. Black Nova Scotian women who live in communities with higher concentrations of Black residents have higher odds of being overdue for cervical screening (odds ratio 1.25; 95% confidence interval 1.19 to 1.30) [7]. Early detection gaps are also evident in breast cancer, since African immigrant women are less likely to receive an early-stage diagnosis, which contributes to poorer survival outcomes [5, 7]. These inequities are particularly concerning given the projected growth of the Black Canadian population from 1.5 million in 2021 to nearly 3.0 million by 2041 [8].

Early detection through cancer screening significantly enhances the chances of successful treatment. Methods such as mammograms for breast cancer, colonoscopies for colorectal cancer, and Pap smears for cervical cancer are crucial for early identification, making prevention efforts vital given the severity of the disease. In Ontario, organized screening programmes for these cancers have been widely implemented, resulting in a reduction in cancer incidence and mortality [9]. However, participation remains uneven across racial, immigrant, and socioeconomic groups. For example, North African women in Ontario are screened for cervical cancer at lower rates compared to non-immigrant women [10], and low-income and newcomer populations continue to experience structural barriers to screening participation [5, 9]. These disparities likely reflect broader social, cultural, and systemic barriers rather than individual unwillingness to participate in screening.

Despite the disproportionate burden experienced by Black communities, the Canadian evidence base remains fragmented and limited in its explicit examination of race-based disparities. Many studies generalize findings across diverse racial groups or focus on populations outside Canada, which overlooks the unique social, historical, and cultural contexts of Black Canadians [11]. As a result, there is limited synthesis of what is known about disparities in cancer screening participation among Black people in Canada, including reported screening rates, barriers, facilitators, and evaluated interventions.

In this review, the term “Black people” refers to individuals who are identified as Black within the included studies, including but not limited to African, Caribbean, and other Black-identifying populations in Canada. This definition is based on how race was reported and operationalised in the primary studies, which often relied on self-identification or administrative classification. We acknowledge that race is a socially constructed concept rather than a biological category; however, it remains an important lens for examining health inequities shaped by systemic and structural factors, including racism and differential access to healthcare [12–14]. Accordingly, the use of “Black” in this review reflects a shared social and structural positioning within the Canadian context rather than a single ethnic or ancestral group, while recognising the diversity within populations classified as Black.

This scoping review therefore synthesises current evidence on disparities in cancer screening participation among Black people in Canada. Specifically, it maps existing evidence, identifies barriers and facilitators, and highlights gaps that require attention. By centring Black Canadians within the analysis, this review aims to inform equity-oriented cancer prevention strategies and support efforts to ensure equal access to preventive healthcare services within Canada’s universal health system.

## Methods

This scoping review followed Arksey and O’Malley’s five-stage methodological framework to systematically map existing evidence on cancer screening among Black populations in Canada [15]. Reporting was guided by the PRISMA Extension for Scoping Reviews (PRISMA-ScR). Each stage of the framework is described below to illustrate how the review was conducted.

### Identifying the research question

In Stage 1, we first established the key research question that guided this review. The primary research question was: “What is known from the existing literature about cancer screening practices, barriers, and facilitators among Black people in Canada?” This question was informed by the Population–Concept–Context (PCC) framework, where the population was Black people in Canada (as defined within the included studies, based on self-identification or administrative classification, including but not limited to African, Caribbean, and other Black-identifying populations), the concept was cancer screening, and the context was the Canadian healthcare setting. Race was considered in this review as a social and structural determinant of health rather than a biological category, reflecting its relevance in shaping health inequities through systemic and institutional processes [12, 14]. The research question clarified the scope of the review and ensured alignment between the study objective and methodological approach.

### Identifying relevant studies

Stage 2 involved designing and executing a comprehensive strategy to identify relevant studies. We systematically searched Sociological Abstracts, SocINDEX, PsychINFO, Embase, Social Science Citation Index, Social Work Abstracts, CINAHL, Scopus, Gender Studies Database, PubMed, ProQuest, and Web of Science. Keywords included “cancer screening,” “Black,” “BIPOC,” and “Canada,” along with terms capturing the intersection of Black populations, cancer, and screening to ensure comprehensive coverage.

We also screened the reference lists of included articles and the libraries of relevant organizations. A research librarian assisted in refining the search strategy, and search results were managed through Covidence.org [16]. The search was conducted on January 17, 2024, and identified 601 articles, of which 257 were duplicates. No publication date restrictions were applied in order to capture the full scope of available evidence. The full search strategy is available in Supplementary File 1.

### Study selection

Stage 3 consisted of a structured two-phase screening process to identify eligible studies. Two research assistants independently screened articles using the inclusion and exclusion criteria.

#### Inclusion criteria

Studies were included if they:

1. Focused on populations identified as Black in Canada, as defined within the individual studies (including but not limited to African, Caribbean, and other Black-identifying populations);
2. Examined cancer screening (including breast, cervical, colorectal, prostate, lung, or anal screening);
3. Reported primary research findings using qualitative, quantitative, or mixed-methods approaches;
4. Were published in English; and.
5. Appeared in peer-reviewed journals.

#### Exclusion criteria

Studies were excluded if they were literature reviews, commentaries, editorials, letters, dissertations, or grey literature; if they did not focus on Black people in Canada; if they did not specifically address cancer screening; if the full text could not be retrieved despite reasonable efforts; or if they did not present primary data.

**Fig. 1:**
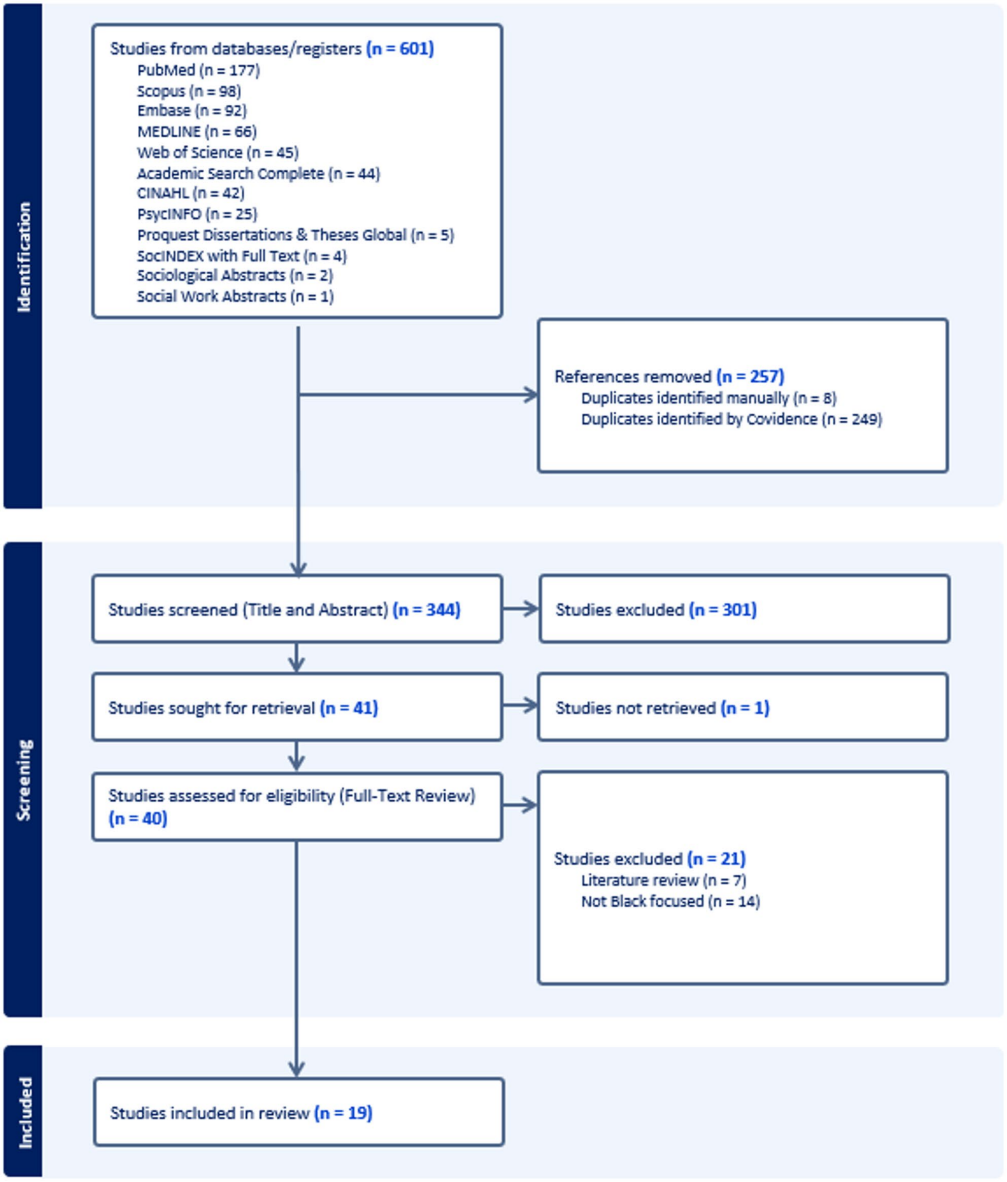
PRISMA Flow diagram for article selection

Phase 1 involved title and abstract screening of 344 records, resulting in 301 exclusions. The remaining 41 articles progressed to Phase 2 for full-text review. During this stage, 22 articles were excluded because they could not be retrieved (*n* = 1), were not focused on Black populations in Canada (*n* = 14), or were literature reviews (*n* = 7). Ultimately, 19 studies met all eligibility criteria and were included for data extraction. A PRISMA flow diagram (Fig. 1) provides a visual summary of the selection process. Disagreements at both screening stages were resolved through discussion, with final adjudication by a senior member of the research team where necessary to achieve consensus. The included studies were published between 2002 and 2023, reflecting over two decades of research in this area.

### Charting the data

Stage 4 focused on systematically extracting and organizing key information from the included studies. Two independent reviewers extracted data into a structured Microsoft Excel template, which was cross-checked by a senior researcher for accuracy and completeness. Extracted data included: province, study population, research setting, study purpose, research questions, methods, study design, sample size, sampling technique, type of cancer screened, screening methods, screening rates, key findings, factors influencing screening, interventions, outcomes, recommendations, and policy implications. Detailed extracted data are provided in the Supplementary Materials.

### Collating, summarizing, and reporting the results

Stage 5 involved synthesizing and summarizing the extracted data to map the range and nature of the research. A narrative synthesis approach was used to identify recurring themes, patterns, and gaps in the literature related to cancer screening among Black people in Canada. Findings were synthesised across cancer types, reported screening outcomes, and recurrent structural, cultural, and socioeconomic barriers and facilitators. This review adhered strictly to Arksey and O’Malley’s five-stage framework throughout [15].

## Results

The findings of this scoping review reveal significant disparities in cancer screening practices among Black people in Canada. Nineteen peer-reviewed studies published between 2002 and 2023 were included. Most studies (58%) focused on cervical cancer screening [7, 9, 10, 17–24], while fewer focused on breast cancer (26%) [9, 22, 25–27], colorectal cancer (21%) [9, 21, 28, 29], and prostate cancer (16%) [30–32]. Anal cancer was investigated in only one study (5%) [23]. This distribution underscores the need for a broader exploration of cancers disproportionately affecting Black people in Canada, such as prostate and colorectal cancers.

The geographic distribution of the studies highlights a concentration in Ontario (11 studies) [9, 10, 17, 18, 21–23, 25, 27, 33], followed by Nova Scotia (3 studies) [7, 19, 29], Alberta (2 studies) [28, 31], and Quebec (1 study) [26]. Two studies were conducted nationwide [20, 30]. This uneven distribution highlights limited evidence from several provinces and territories and suggests a need for more geographically diverse research.

Synthesizing these findings reveals several recurring themes:

- Barriers: Socioeconomic challenges, cultural stigmas, and structural racism were the most frequently cited obstacles to screening [11, 13, 22, 24, 33, 34]. For instance, Black immigrant women reported lower cervical cancer screening rates compared to other groups, often due to financial and language barriers [24, 35].
- Facilitators: Tailored interventions, such as culturally sensitive education and the use of HPV self-sampling kits, were effective in improving participation rates [13, 25, 29, 35].
- Research Gaps: Limited studies investigated advanced screening technologies or community-specific interventions targeting rural and underserved areas.

The supplementary data extraction tables summarise key study characteristics, objectives, key findings and recommendations (see Supplementary Table 1).

### Participants characteristics

Included studies examined diverse Black communities and related populations across Canada, including African, Caribbean, and African-Caribbean-Black (ACB) populations, as well as studies in which Black participants were analyzed within broader racialized or immigrant populations. Study populations included ACB women living with HIV in Toronto [17] diverse ethnic groups of Canadian men for PSA screening [30, 31], and individuals aged 50–74 in Calgary for colorectal cancer (CRC) screening [28] Specific attention was given to under- or never-screened women of South or West Asian, Middle Eastern, or North African ancestry in the Greater Toronto Area [22] and men in Calgary, particularly Black and Métis individuals, for PSA testing [31] Caribbean immigrant women in Ontario were also highlighted for cervical cancer screening [18] alongside women in Cape Breton Island, Nova Scotia, for Pap smear screening [19]. In another report, immigrant women in Ontario, particularly from Muslim-majority and non-Muslim-majority countries, Haitian immigrant women in Quebec for mammography [26] and diverse ethnic groups in Nova Scotia for CRC screening were studied [29]. The overarching aim of these studies was to identify and address sociodemographic disparities and promote equitable access to preventive healthcare services among various ethnic, immigrant, and socioeconomically diverse groups in Canada.

Table 1 shows that evidence on cancer screening among Black populations in Canada is concentrated in Ontario and primarily focused on cervical cancer, with fewer studies addressing prostate and colorectal screening despite their documented burden. Most studies were observational, with relatively few rigorously evaluated interventions.

**Table 1:** Characteristics and key findings of included studies on cancer screening among black populations in Canada

Author (Year)	Province	Study design	Population (*N*)	Cancer type	Outcome measure	Key findings	Intersectional factors	Barriers	Facilitators	Intervention
Andany et al. (2014) [17]	ON (Toronto)	Cross-sectional survey	ACB women living with HIV (126)	Cervical	Self-reported Pap testing (past year, past 3 years)	Screening relatively high, but under-screening persisted; higher screening with longer time in Canada and recent primary care contact	Age, income, education, immigration/time in Canada, healthcare access	Not knowing where to go, perceived lack of need, stigma, recent immigration	Family doctor contact, longer time in Canada, higher income/education	None
Beaulac et al. (2006) [30]	Canada-wide	Population survey analysis	Men aged 50 + without prostate cancer (18,884)	Prostate	Ever PSA and recent PSA testing	PSA screening common; lower uptake among immigrants, those not speaking English/French, rural residents, lower income	Age, income, education, immigration, language, rurality, primary care access	Language barriers, no family doctor, low income, rural residence	Regular primary care, higher education/income, more medical visits	None
Crouse et al. (2015) [28]	AB (Calgary)	Programme evaluation/observational (administrative)	Adults 50–74 (27,572)	Colorectal	FIT participation rates	Lower FIT uptake in Black residents, Indigenous groups, and recent immigrants; geographic hot/cold spots	Immigration, ethnicity, education, neighbourhood, access	Limited primary care attachment, immigration-related barriers	Neighbourhood access to distributing providers, programme availability	Calgary community-based FIT programme (existing)
Devotta et al. (2023) [10]	ON (GTA)	Qualitative (CFIR-guided)	Under/never screened women 30–69 (108), incl. North African	Cervical	Acceptability and uptake of HPV self-sampling	HPV self-sampling highly acceptable; privacy and convenience increased willingness	Immigration, language, cultural/religious identity	Stigma, limited English, uncertainty about test, negative clinical experiences	Privacy, convenience, community champions	HPV self-sampling (implementation-focused)
Gillis et al. (2020) [33]	ON	Cohort/clinic-based observational	Men with HIV (1,677)	Anal	Ever screened (DARE/anal cytology/anoscopy)	ACB men less likely to be screened than White men	Race/ethnicity, sexual orientation, engagement in care	Limited access to anoscopy, communication gaps, guideline uncertainty	Comfort discussing anal health, longer engagement in HIV care	None
Gorday et al. (2014) [31]	AB (Calgary)	Administrative/census-linked observational	Men in Calgary (75,914)	Prostate	PSA testing patterns	Visible Minority Black and Métis men less likely to receive PSA; geographic variation	Ethnicity, income, education, neighbourhood	Lower SES, weaker access to primary care	Higher income areas, stronger primary care attachment	None
Hyman et al. (2002) [36]	ON (Toronto/Hamilton)	Administrative data analysis	Physicians (5,062) and patients (1,116,162)	Cervical	Cervical screening rates (administrative)	Physician characteristics influenced screening rates	Provider factors (sex, training, years)	Practice-related differences	Some practice models associated with better screening	None
Johnston et al. (2003) [19]	NS (Cape Breton)	Controlled intervention study	Women 25–69 (22,836)	Cervical	Pap uptake after reminder	Reminder letters increased Pap uptake, especially among under/never screened	Age, income, rurality, prior screening	Under-screened status, low income, rural access issues	Reminder prompts, health system contact	Reminder letter intervention
Johnston et al. (2004) [7]	NS	Population-based observational	Women 18+ (360,587)	Cervical	Recent Pap smear (community-level predictors)	Lower Pap uptake in low-income, rural, Aboriginal and mixed Black communities	Community ethnicity, income, rurality, age	Rural residence, low-income communities, mixed Black community composition	Higher-income areas, younger age groups	None
Lee et al. (2019) [20]	ON (Toronto/GTA)	EMR + administrative observational	Women 50–74 (53,971)	Breast	Up-to-date mammography	Demonstrated racial screening inequities; network data can identify gaps	Race/ethnicity, access	Inequities by racial group	System-level identification of inequities	None
Lofters et al. (2017) Ko-Pamoja [21]	ON (Toronto/GTA)	Pilot intervention evaluation	Black women (30)	Breast and cervical	Knowledge/self-efficacy; screening completion follow-up	Improved knowledge and self-efficacy; some overdue women completed screening soon after	Race, gender, immigration, neighbourhood disadvantage	Fear, low health literacy, transport/access challenges	Afrocentric delivery, lay educators, church setting, peer support	Ko-Pamoja lay health educator-led Afrocentric programme
Lofters et al. (2017) Muslim-majority proxy [22]	ON	Population-based cohort/administrative	Immigrant women 21–69 (761,019)	Cervical	Up-to-date Pap testing	Lower Pap uptake among women born in Muslim-majority countries; highest overdue among Sub-Saharan Africa Muslim-majority origin	Country/region of origin, language, income, primary care model	Language, lower income, male physician, lack of attachment	Female physician, certain primary care models	None
Lofters et al. (2017) SDOH in primary care [25]	ON (Toronto)	Cross-sectional observational (primary care)	Adult primary care patients (5,766)	Breast, cervical, colorectal	Up-to-date screening (multiple)	Individual-level income/housing better detected inequities than neighbourhood measures	Income, housing, immigration, language	Low income, unstable housing, incomplete SES data	Home ownership, stable SES, primary care supports	None
Lorentz et al. (2018) MORE programme [32]	ON (Toronto)	Descriptive programme study	High-risk men (270)	Prostate	Participation in high-risk screening/monitoring	High-risk clinic supports screening/monitoring for WA/C ancestry and genetic risk	Ancestry, family history, genetics, access	Unequal access to high-risk services; inconsistent guidance	Structured clinic pathway, specialist access	MORE high-risk screening programme
McDonald & Kennedy (2007) [23]	ON (Toronto)	Population survey analysis	Immigrant and minority women (sample size as reported)	Cervical	Pap testing prevalence	Immigrant and minority women had lower Pap uptake; uptake improved with longer time in Canada but remained uneven	Immigration, years in Canada, language, ethnicity, education	Language, modesty norms, system unfamiliarity	Longer time in Canada, education, urban residence	None
Nnorom et al. (2021) TAIBU [9]	ON (Toronto)	Quality improvement/programme evaluation	Patient charts (708)	Breast, cervical, colorectal	Screening offers and uptake over time	Large increases in screening offers over time; community co-design and Afrocentric approach central	Race, immigration, SES, racism experiences	Fear, stigma, low trust, low income, oppression/system barriers	Culturally tailored education, reminders, audits, community engagement	Afrocentric QI programme at TAIBU CHC
Raynault et al. (2020) [26]	QC (Montreal)	Qualitative study	Haitian immigrant women (32)	Breast	Understanding of referral letter; literacy barriers	Major difficulty interpreting programme letter; literacy/language barriers reduced engagement	Immigration, language, literacy, neighbourhood disadvantage	Low literacy, limited French/English, mail-system unfamiliarity	Creole communication, community support, simplified materials	None (recommendations for communication redesign)
Sullivan et al. (2022) [29]	NS	Programme data analysis	FIT participants 50–74 (208,702)	Colorectal	FIT participation by race/ethnicity	Lower FIT participation among Black/African Canadian vs. White	Race/ethnicity, age, sex	Lower engagement with mailed FIT programme	Universal programme structure (but uneven uptake)	Provincial FIT programme (existing)
Vahabi et al. (2016) [27]	Ontario	Retrospective population-based cohort study using linked administrative health databases	Immigrant women aged 50–69 (*N* = 183,332)	Breast cancer	Mammography uptake within a 2-year screening period	Screening rate was 57.1%, with disparities by region of origin. Rates were highest among Caribbean/Latin American women and lowest among South Asian women. Higher income, longer time in Canada, primary care enrollment, and female physicians increased screening, while recent immigration and low income were associated with lower uptake	Region of origin, immigration status (recent vs. established), income, language proficiency, healthcare access, physician characteristics (gender, training), and length of stay in Canada	Language barriers, low health literacy, low income, recent immigration/refugee status, limited healthcare access, lack of periodic health examinations, male or internationally trained physicians, and system navigation challenges	Higher income, longer time in Canada, enrollment in primary care models (e.g., Family Health Teams), having a female physician, regular healthcare visits, and ethnic concordance between patient and physician	None

Across cancer types and provinces, a consistent pattern of under-screening or delayed adherence among Black communities was observed. These disparities were closely linked to intersecting structural and socioeconomic factors, including low income, immigration status, language barriers, housing instability, rural residence, and limited attachment to primary care. Importantly, inequities were repeatedly attributed to systemic and structural barriers rather than individual unwillingness to participate in screening.

Several studies identified promising strategies to improve screening participation. Culturally tailored, community-based programmes, Afrocentric approaches, reminder systems, and HPV self-sampling were associated with improved knowledge, engagement, and, in some cases, increased screening uptake. However, intervention research remains limited, and evidence from several provinces and rural settings is sparse.

Overall, the evidence indicates that cancer screening disparities among Black people in Canada are persistent, structurally patterned, and intersectional, with emerging but insufficiently evaluated strategies to reduce inequities.

### Cancer screening rates

There were notable disparities in cancer screening rates among Black individuals in Canada. The included studies revealed lower rates of Pap smear testing among Black immigrant women compared to Canadian-born women and other immigrant groups, despite high intentions expressed by physicians [18]. Actual screening rates among Caribbean patients, including Black immigrants, often fell below estimated figures, indicating a gap between intention and practise [17, 18, 33]. Moreover, the study examining HIV-positive African-Caribbean Black individuals indicated relatively high rates of recent Pap testing, but a portion of the population remained overdue or had never had a Pap test, suggesting ongoing challenges in access or awareness [17]. Across the studies, these disparities were consistently linked to intersecting social, cultural, and systemic barriers rather than individual hesitancy alone.

Additionally, disparities in prostate cancer screening are evident, with individuals identifying as “Visible Minority Black” being less likely to receive PSA tests compared to the general population, which again indicated ethnic disparities in access to screening services [31]. Furthermore, disparities extended to participation rates in various cancer screening programmes, with notable differences observed among Haitian immigrant women in Montreal [26], reflecting language, literacy, and system navigation challenges [30]. Overall, the findings demonstrate a persistent pattern of under-screening among Black populations across cancer types and across provinces.

### Interventions and recommendations identified in the literature

#### Physician awareness and patient education

This review identified several challenges in the implementation of health interventions and offered recommendations to overcome these obstacles. Increasing physician awareness and patient education were crucial aspect in terms of addressing cancer screening discrepancies among Black people in Canada. Specifically, allocating more time for patient education during visits has been recommended to ensure Black patients understand the importance of regular screenings and how to access these services, particularly for cervical cancer risks and screening guidelines [9, 18]. Additionally, offering culturally sensitive training to healthcare providers was emphasized as essential for improving communication and trust between physicians and Black patients, thereby increasing screening participation rates [9, 17, 18, 23, 28]. Studies consistently indicated that provider–patient communication remains a key determinant of screening participation.

#### Reminder systems and access to screening services

Implementing reminder systems for physicians and patients emerged as a key recommendation to ensure timely screenings. Such systems were shown to be effective in maintaining high screening rates among Black communities [25, 28]. Additionally, improving access to screening services through mobile units, community clinics, and partnerships with local providers was highlighted as crucial [25, 32]. These initiatives make screenings more accessible, especially in underserved areas [25, 32]. These structural supports were shown to narrow screening gaps when implemented consistently.

#### Tailored education and outreach

Developing culturally specific educational programmes tailored to the Black community was identified as a significant strategy for enhancing cancer screening rates. These programmes addressed unique cultural needs and concerns, thus increasing their effectiveness [9, 17, 18, 23, 28] Furthermore, focusing on raising awareness among high-risk Black groups through community-specific interventions was shown to be effective. This targeted approach ensured the most vulnerable populations receive the necessary information and encouragement to participate in screening programmes [7, 28]. Culturally grounded approaches were among the most consistently effective strategies across studies.

#### Addressing socioeconomic barriers

The studies highlighted the need to provide financial assistance and transportation support to overcome socioeconomic barriers faced by Black communities. These supports ensure equitable access to screenings, regardless of financial status [17, 20, 25, 27, 28, 33]. Ensuring the accessibility of screening programmes for Black individuals, regardless of their financial status, was also emphasized [17, 23, 26–28]. Socioeconomic supports were identified not as optional but essential components of equitable screening programs.

#### Utilization of accessible screening technologies

The incorporation of advanced screening technologies, such as Human Papilloma Virus (HPV) self-sampling, was recommended to make cancer screenings more accessible and less invasive for Black patients. This innovative approach has been shown to increase participation rates, particularly among those hesitant to undergo traditional methods [10]. Self-sampling demonstrated strong acceptability among under-screened women and represents a promising tool for reducing inequities.

#### Community engagement

The findings of this review underscored the importance of implementing regular audits of educational programmes to ensure they remain effective and relevant to the Black community. These audits help identify areas for improvement and ensure the efficient use of resources [9]. Additionally, gathering community feedback was identified as crucial for the ongoing effectiveness of cancer screening programmes. Engaging with the Black community helps healthcare providers understand their specific needs and concerns, leading to more tailored and effective interventions [9]. Community co-design was repeatedly highlighted as a critical factor for program success.

#### Policy strategies for enhancing cancer screening equity

Key policy strategies included standardizing screening guidelines, revising reimbursement policies, improving healthcare access, and enhancing cultural competency training [9, 25]. Specific recommendations for PSA screening included clearer guidelines and expanded primary care access [18, 33]. Recommendations for colorectal cancer emphasized culturally tailored interventions and broad access to screening kits [20, 32]. For immigrant women, strengthening primary care access and developing tailored programs were suggested [22, 25, 29]. Across studies, policy-level changes were viewed as necessary to ensure long-term, sustainable improvements in screening equity.

## Discussion

This scoping review set out to answer the question: “What is known from the existing literature about cancer screening practices, barriers, and facilitators among Black people in Canada?” Overall, the findings show a consistent pattern of under-screening and later-stage diagnosis among Black populations across several cancer types, despite the existence of publicly funded screening programs. These results align with national registry–census linkage studies that report higher mortality from breast, colorectal, prostate, and pancreatic cancers in Black patients compared with non-Black patients in Canada, even after accounting for social determinants of health [4]. Recent work on breast cancer has further shown that Black women in Canada are more likely to be diagnosed at a younger age, with more aggressive subtypes and worse survival than White women, reinforcing the importance of early and equitable access to screening in this population [34].

### Geographic and methodological patterns

The concentration of studies in Ontario, with only a few from Alberta, Nova Scotia, and Quebec, reflects both where large administrative datasets and equity-focused primary care initiatives are located and where Black populations are more densely clustered. However, this geographic skew leaves important gaps in understanding screening practices and barriers in other provinces and territories, particularly in the Prairies and Atlantic Canada where Black communities are smaller but growing. This gap underscores the need for more geographically representative research to inform national equity strategies. This limitation mirrors national concerns that current data systems make it difficult to monitor racial health inequities consistently across jurisdictions [14].

Methodologically, most included studies used cross-sectional or retrospective cohort designs, often relying on administrative data or self-report. Few studies employed longitudinal or rigorously evaluated intervention designs. This is consistent with broader Canadian and international literature on cancer screening among migrants and racialized groups, where observational designs dominate and causal inference is limited [35]. While administrative data enable large-scale, population-level analyses, they frequently lack standardized race and ethnicity variables, which constrains intersectional analyses by race, gender, class, and immigration status. Recent Canadian work has highlighted the urgent need for systematic race-based data collection and equity-focused reporting standards to better monitor and address cancer inequities, including those affecting Black communities [4]. Thus, strengthening race-based data collection remains critical for advancing equity-focused research and policy development.

### Interpreting disparities in cancer screening

The results of this review show that lower screening participation among Black populations is not simply a matter of individual choice, but reflects a multi-layered set of structural, social, and system-level barriers. Studies in Canada and other high-income countries consistently document that immigrants and racialized groups face language barriers, limited health literacy, economic constraints, and difficulties navigating the healthcare system, which are all associated with lower uptake of breast, cervical, and colorectal screening [35]. For Black communities in Canada, these barriers are compounded by experiences of racism and discrimination in healthcare settings, historical mistrust, and policies that have not been designed with Black populations in mind [4].

Our findings on under-screening among Black and immigrant women, Haitian women in Montreal, and Black or visible minority men for prostate and colorectal cancer screening are consistent with these broader patterns. They also align with national analyses showing elevated mortality from prostate cancer and several other cancers among Black adults in Canada, even after adjustment for socioeconomic factors [12]. Taken together, these findings suggest that inequities in screening represent a key pathway through which structural racism and socioeconomic disadvantage contribute to poorer cancer outcomes.

### Interventions and how they fit the wider evidence

The interventions identified in the included studies, such as Afrocentric quality improvement initiatives at TAIBU Community Health Centre and the Ko Pamoja lay health educator program, provide important examples of how community driven approaches can close screening gaps. These programs, which combine culturally tailored education, patient reminders, provider audits, and community leadership, have been shown to substantially increase breast, cervical, and colorectal screening participation among Black and immigrant patients in Toronto [9]. Similar peer education and community outreach initiatives in other Canadian settings have also reported improved cancer-related knowledge and screening intentions among racialized and immigrant women [25].

Taken together, this evidence supports the conclusion that generic “one-size-fits-all” reminder systems are insufficient for addressing inequities. Instead, interventions that explicitly name racism, center Black cultural knowledge, and engage communities as partners rather than passive recipients appear more effective in improving screening rates [37]. The strong acceptability of HPV self sampling among under and never screened women in the included studies is also consistent with international evidence that self sampling and home-based testing can reduce structural and psychosocial barriers for marginalized groups [35].

### Policy and practice implications

From a policy perspective, the findings of this review suggest that improving cancer screening for Black populations in Canada requires changes at multiple levels. At the health system level, there is growing recognition that race and ethnicity should be routinely captured in cancer registries, screening programs, and surveys to enable monitoring of inequities and evaluation of interventions [4]. At the clinical level, expanding access to team-based primary care models and embedding culturally safe navigation and outreach roles has been associated with smaller post-pandemic widening of screening inequities by income and immigration status in Ontario [38].

The review also supports calls to revisit screening guidelines and outreach strategies in light of evidence that Black women in Canada are diagnosed with breast cancer at younger ages and with more aggressive disease, and that Black adults experience distinct patterns of risk and mortality [34]. Policy makers should therefore consider equity-focused adaptations such as targeted, earlier, or more intensive screening outreach for high-risk Black communities, alongside broader investments in primary care access, language-concordant services, and transportation or financial supports [39].

### Strengths, limitations, and future directions

This scoping review provides a comprehensive synthesis of literature on cancer screening disparities among Black people in Canada, highlighting critical barriers, facilitators, and gaps in existing research. By covering multiple cancer types and screening methods, it offers a broad perspective on the challenges faced by this underserved population. A key strength of this review is the structured and transparent use of Arksey and O’Malley’s five-stage framework, which enhances methodological rigor. The explicit focus on intersectional determinants strengthens the analytical depth of the review.

Despite these strengths, several limitations must be acknowledged. Only 19 studies met the inclusion criteria, reflecting both the emerging nature of this field and the limited evidence available. Geographic representation was also uneven, with most studies conducted in Ontario and far fewer in other provinces, which limits the national generalizability of findings. The restriction to English-language publications may have resulted in the exclusion of relevant French-language or multilingual studies, particularly from Quebec or immigrant communities. Many included studies rely on cross-sectional or self-reported data, introducing recall and social desirability bias and limiting causal interpretation. The absence of longitudinal research limits understanding of long-term screening behavior and the sustained impact of interventions. Finally, the focus on peer-reviewed literature may have excluded valuable evidence from grey literature sources.

Future research should prioritize longitudinal and intervention studies that evaluate the sustained impact of Afrocentric and community-led strategies, as well as policy and primary-care innovations designed to reduce screening inequities over time. Studies should also include more detailed analyses of Black subgroups, considering ancestry, gender, age, and immigration pathway to better capture heterogeneity within Black populations. Importantly, future work should be co-designed with Black communities and incorporate race-based data collection aligned with emerging Canadian best practices, supporting a shift from documenting disparities toward implementing and evaluating concrete, equity-focused solutions.

## Conclusion

This scoping review synthesised evidence on cancer screening practices, barriers, and facilitators among Black populations in Canada and highlights persistent inequities across multiple cancer types. Despite universal healthcare coverage, Black communities continue to experience lower screening participation, later-stage diagnoses, and limited access to culturally safe and responsive services. The findings demonstrate that these disparities arise from complex structural, socioeconomic, cultural, and systemic factors rather than individual choice.

Community-driven and culturally tailored interventions, including Afrocentric health promotion, patient navigation, provider training, and accessible technologies such as HPV self-sampling, show promise in increasing engagement and improving screening outcomes. However, the current evidence base is geographically narrow and methodologically limited, underscoring the need for longitudinal, intervention-focused, and equity-oriented research.

Addressing cancer screening inequities requires coordinated action across policy, practice, and community settings. Strengthening race-based data collection, expanding culturally safe primary care models, and investing in community partnerships are essential steps toward improving cancer prevention and early detection for Black populations in Canada. By prioritising equity-oriented strategies and centring the voices and experiences of Black communities, Canada can move toward a more just and effective cancer screening system.

## Supplementary Information

Supplementary Material 1.

Supplementary Material 2.

Supplementary Material 3.

## Data Availability

All data generated or analyzed during this study are included in this published article [and its supplementary information files].

## Abbreviations

ACB: African-Caribbean-Black
CS: Cancer Screening
CHS: Community Health Sciences
CME: Continuing Medical Education
CRC: Colorectal Cancer
CCT: Cultural Competency Training
FIT: Fecal Immunochemical Test
HPV: Human Papilloma Virus
PCP: Primary Care Physician
PSA: Prostate-Specific Antigen
SES: Socioeconomic Status
WHO: World Health Organization
PST: Pap Smear Test
