# Supplementary material for: Cancer screening discrepancies among Black people in Canada: a scoping review
Source: BMC Public Health. 2026 Apr 22;26:1802. doi: 10.1186/s12889-026-27317-0 (PMC13235013; doi:10.1186/s12889-026-27317-0)

Scoping Review: Cancer Screening for Black Canadians

Search Strategies: Caitlin McClurg, MLIS (Libraries and Cultural Resources, University of Calgary)

January 17 2024

Ovid MEDLINE(R) ALL <1946 to January 16, 2024>

<https://ezproxy.lib.ucalgary.ca/login?url=https://ovidsp.ovid.com/ovidweb.cgi?T=JS&NEWS=N&PAGE=main&SHAREDSEARCHID=6jJ7p68v1XT3T4lGljxr3EysPKGKcSHSSTob2ibWAMAmY6P5BwoC5nUxBdOe4YPDz>

1 exp "Early Detection of Cancer"/ 39224

2 (cancer* adj2 detect*).tw,kf. 35363

3 (cancer* adj2 screen*).tw,kf. 49278

4 exp Mass Screening/ 145109

5 screen*.tw,kf. 1013168

6 1 or 2 or 3 or 4 or 5 1088030

7 exp Neoplasms/ 3922958

8 neoplasm*.tw,kf. 318600

9 cancer*.tw,kf. 2337508

10 malignan*.tw,kf. 706696

11 7 or 8 or 9 or 10 4756224

12 exp "Black or African American"/ 62820

13 exp Racial Groups/ 106362

14 racialized.tw,kf. 1275

15 Caribbean.tw,kf. 18883

16 African.tw,kf. 175527

17 Afro-Canadian.tw,kf. 4

18 African Canad*.tw,kf. 59

19 BIPOC.tw,kf. 364

20 (Caribbean adj2 Canad*).tw,kf. 29

21 Jamaican.tw,kf. 1808

22 Black.tw,kf. 176867

23 12 or 13 or 14 or 15 or 16 or 17 or 18 or 19 or 20 or 21 or 22 407860

24 exp Canada/ 184583

25 Canad*.tw,kf. 166781

26 exp British Columbia/ 11608

27 British Columbia.tw,kf. 11263

28 exp Alberta/ 9435

29 Alberta.tw,kf. 11867

30 exp Saskatchewan/ 2784

31 Saskatchewan.tw,kf. 3522

32 exp Manitoba/ 3607

33 Manitoba.tw,kf. 4505

34 exp Ontario/ 32268

35 Ontario.tw,kf. 36079

36 exp New Brunswick/ 795

37 New Brunswick.tw,kf. 1418

38 exp Nova Scotia/ 2720

39 Nova Scotia.tw,kf. 3056

40 exp "Newfoundland and Labrador"/ 1501

41 Newfoundland.tw,kf. 2033

42 exp Yukon Territory/ 250

43 Yukon.tw,kf. 772

44 exp Northwest Territories/ 439

45 Northwest Territories.tw,kf. 654

46 exp Nunavut/ 381

47 Nunavut.tw,kf. 651

48 exp Prince Edward Island/ 324

49 Prince Edward Island.tw,kf. 630

50 24 or 25 or 26 or 27 or 28 or 29 or 30 or 31 or 32 or 33 or 34 or 35 or 36 or 37 or 38 or 39 or 40 or 41 or 42 or 43 or 44 or 45 or 46 or 47 or 48 or 49 275572

51 6 and 11 and 23 and 50 66

Embase <1974 to 2024 January 16>

<https://ezproxy.lib.ucalgary.ca/login?url=https://ovidsp.ovid.com/ovidweb.cgi?T=JS&NEWS=N&PAGE=main&SHAREDSEARCHID=2Lui09sHOuBRXsoOIQvuSpnfwFQh5rXza9mm8lbewCdemGRaSWzSvrBMliIVOX2T3>

1 exp cancer screening/ 100205

2 screen*.tw,kf. 1422929

3 (cancer* adj2 detect*).tw,kf. 50607

4 (cancer adj2 screen*).tw,kf. 70484

5 1 or 2 or 3 or 4 1478375

6 exp malignant neoplasm/ 4311692

7 cancer*.tw,kf. 3313474

8 neoplasm*.tw,kf. 331851

9 malignan*.tw,kf. 1008115

10 6 or 7 or 8 or 9 5561558

11 exp Black person/ 148842

12 exp racial identity/ 418

13 Black.tw,kf. 229509

14 exp "Caribbean (person)"/ 3209

15 Caribbean.tw,kf. 22021

16 exp Jamaican/ 377

17 exp African/ 40430

18 racialized.tw,kf. 1262

19 Caribbean.tw,kf. 22021

20 Jamaican.tw,kf. 2105

21 African.tw,kf. 234704

22 BIPOC.tw,kf. 457

23 Afro-Canadian.tw,kf. 5

24 (Caribbean adj2 Canad*).tw,kf. 40

25 (African adj2 Canad*).tw,kf. 141

26 11 or 12 or 13 or 14 or 15 or 16 or 17 or 18 or 19 or 20 or 21 or 22 or 23 or 24 or 25 521931

27 exp Canada/ 219756

28 Canad*.tw,kf. 232434

29 exp British Columbia/ 5227

30 British Columbia.tw,kf. 14738

31 exp Alberta/ 3457

32 Alberta*.tw,kf. 16620

33 exp Saskatchewan/ 1203

34 Saskatchewan.tw,kf. 4065

35 Manitoba.tw,kf. 5637

36 exp Manitoba/ 1683

37 exp Ontario/ 14003

38 Ontario.tw,kf. 47176

39 exp New Brunswick/ 484

40 New Brunswick.tw,kf. 1674

41 exp Nova Scotia/ 1258

42 Nova Scotia.tw,kf. 3982

43 exp Prince Edward Island/ 218

44 Prince Edward Island.tw,kf. 666

45 exp "Newfoundland and Labrador"/ 702

46 Newfoundland.tw,kf. 2401

47 exp Yukon/ 191

48 Yukon.tw,kf. 807

49 exp Northwest Territories/ 171

50 Northwest Territories.tw,kf. 711

51 exp Nunavut/ 231

52 Nunavut.tw,kf. 709

53 27 or 28 or 29 or 30 or 31 or 32 or 33 or 34 or 35 or 36 or 37 or 38 or 39 or 40 or 41 or 42 or 43 or 44 or 45 or 46 or 47 or 48 or 49 or 50 or 51 or 52 341000

54 5 and 10 and 26 and 53 92

APA PsycInfo <1806 to January Week 2 2024>

<https://ezproxy.lib.ucalgary.ca/login?url=https://ovidsp.ovid.com/ovidweb.cgi?T=JS&NEWS=N&PAGE=main&SHAREDSEARCHID=E2L5rA3cxWRyL7JS9uOLzicCagFOPr5qCcC9sMdShPnBnAn3XHOKPFbEXID5gPm0>

1 exp Cancer Screening/ 5714

2 (cancer adj2 detect*).tw,id. 811

3 (cancer adj2 screen*).tw,id. 5750

4 screen*.tw,id. 124631

5 1 or 2 or 3 or 4 125599

6 exp Neoplasms/ 62962

7 neoplasm*.tw,id. 2736

8 cancer*.tw,id. 75462

9 malignan*.tw,id. 7888

10 6 or 7 or 8 or 9 89463

11 exp Blacks/ 61323

12 Black.tw,id. 75624

13 exp Ethnic Identity/ 19522

14 racialized.tw,id. 3975

15 exp "Racial and Ethnic Groups"/ 160939

16 exp Caribbean Cultural Groups/ 599

17 exp African Cultural Groups/ 3987

18 Caribbean.tw,id. 4670

19 African.tw,id. 76819

20 Jamaican.tw,id. 869

21 exp "People of Color"/ 1022

22 BIPOC.tw,id. 383

23 11 or 12 or 13 or 14 or 15 or 16 or 17 or 18 or 19 or 20 or 21 or 22 248297

24 Canad*.tw,id. 58287

25 British Columbia.tw,id. 2632

26 Alberta.tw,id. 2261

27 Saskatchewan.tw,id. 570

28 Manitoba.tw,id. 878

29 Ontario.tw,id. 8203

30 New Brunswick.tw,id. 289

31 Nova Scotia.tw,id. 619

32 Prince Edward Island.tw,id. 80

33 Newfoundland.tw,id. 405

34 Yukon.tw,id. 86

35 Northwest Territories.tw,id. 96

36 Nunavut.tw,id. 120

37 24 or 25 or 26 or 27 or 28 or 29 or 30 or 31 or 32 or 33 or 34 or 35 or 36 64014

38 5 and 10 and 23 and 37 25

CINAHL Plus with Full Text 42 results

detect* OR screen* [Select a Field]

**AND** cancer* OR malignan* OR neoplasm* [Select a Field]

**AND** Black OR BIPOC OR African OR Caribbean OR Jamaican[Select a Field]

**AND** Canad* OR Alberta OR British Columbia OR Saskatchewan OR Manitoba OR Ontario OR New Brunswick OR Nova Scotia OR Prince Edward Island OR Newfoundland OR Yukon OR Northwest Territories OR Nunavut [Select a Field]


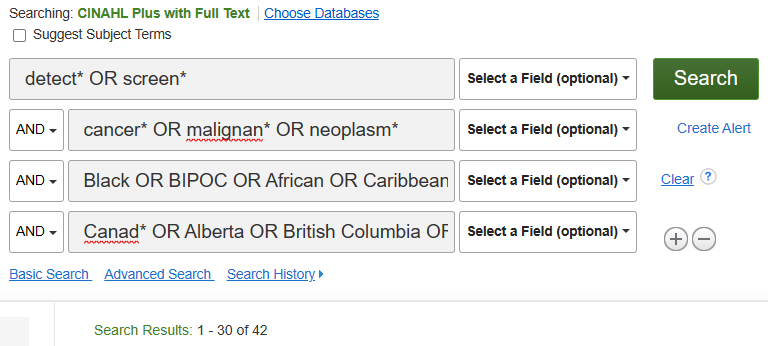


Pubmed 177 results

((cancer*[Title/Abstract] OR malignan*[Title/Abstract] OR neoplasm*[Title/Abstract]) AND (Black[Title/Abstract] OR BIPOC[Title/Abstract] OR African[Title/Abstract] OR Caribbean[Title/Abstract] OR Jamaican[Title/Abstract])) AND (Canad*[Title/Abstract] OR Alberta[Title/Abstract] OR British Columbia[Title/Abstract] OR Saskatchewan[Title/Abstract] OR Manitoba[Title/Abstract] OR Ontario[Title/Abstract] OR New Brunswick[Title/Abstract] OR Nova Scotia[Title/Abstract] OR Prince Edward Island[Title/Abstract] OR Newfoundland[Title/Abstract] OR Yukon[Title/Abstract] OR Northwest Territories[Title/Abstract] OR Nunavut[Title/Abstract])

Academic Search Complete 44 results

detect* OR screen* [Select a Field]

**AND** cancer* OR malignan* OR neoplasm* [Select a Field]

**AND** Black OR BIPOC OR African OR Caribbean OR Jamaican[Select a Field]

**AND** Canad* OR Alberta OR British Columbia OR Saskatchewan OR Manitoba OR Ontario OR New Brunswick OR Nova Scotia OR Prince Edward Island OR Newfoundland OR Yukon OR Northwest Territories OR Nunavut [AB Abstract or Author-Supplied Abstract]


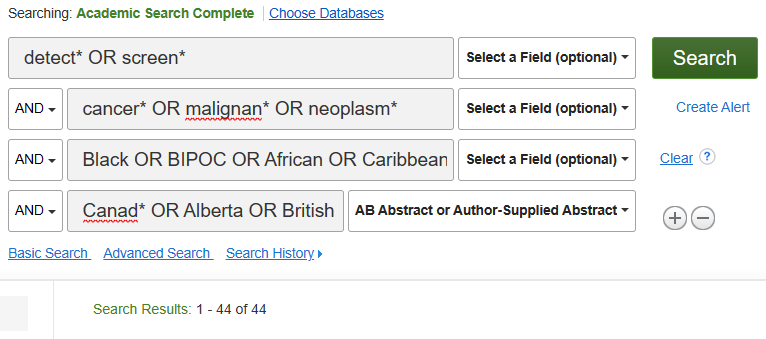


Social Work Abstracts 1 result

cancer* OR malignan* OR neoplasm* [Select a Field]

**AND** Black OR BIPOC OR African OR Caribbean OR Jamaican[Select a Field]

**AND** Canad* OR Alberta OR British Columbia OR Saskatchewan OR Manitoba OR Ontario OR New Brunswick OR Nova Scotia OR Prince Edward Island OR Newfoundland OR Yukon OR Northwest Territories OR Nunavut [Select a Field]

WebofScience 45 results


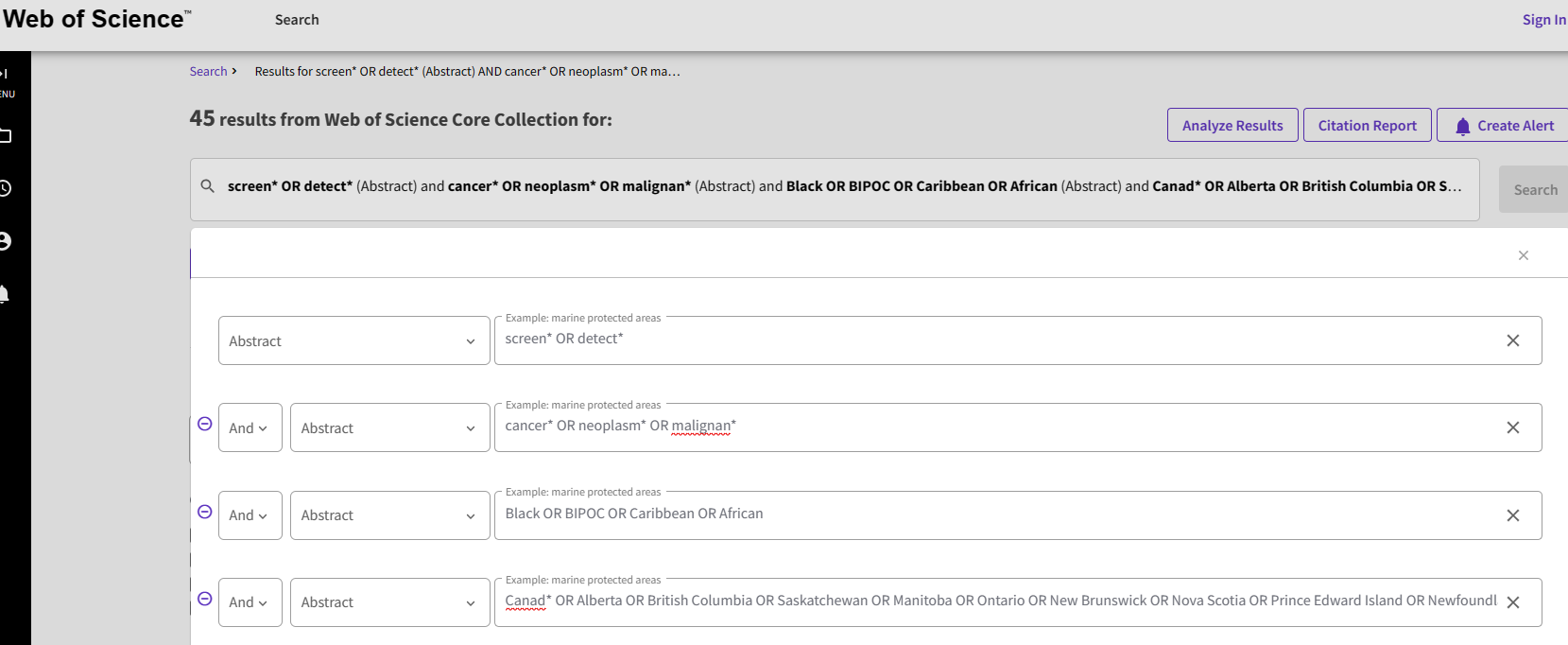


Scopus 98 results


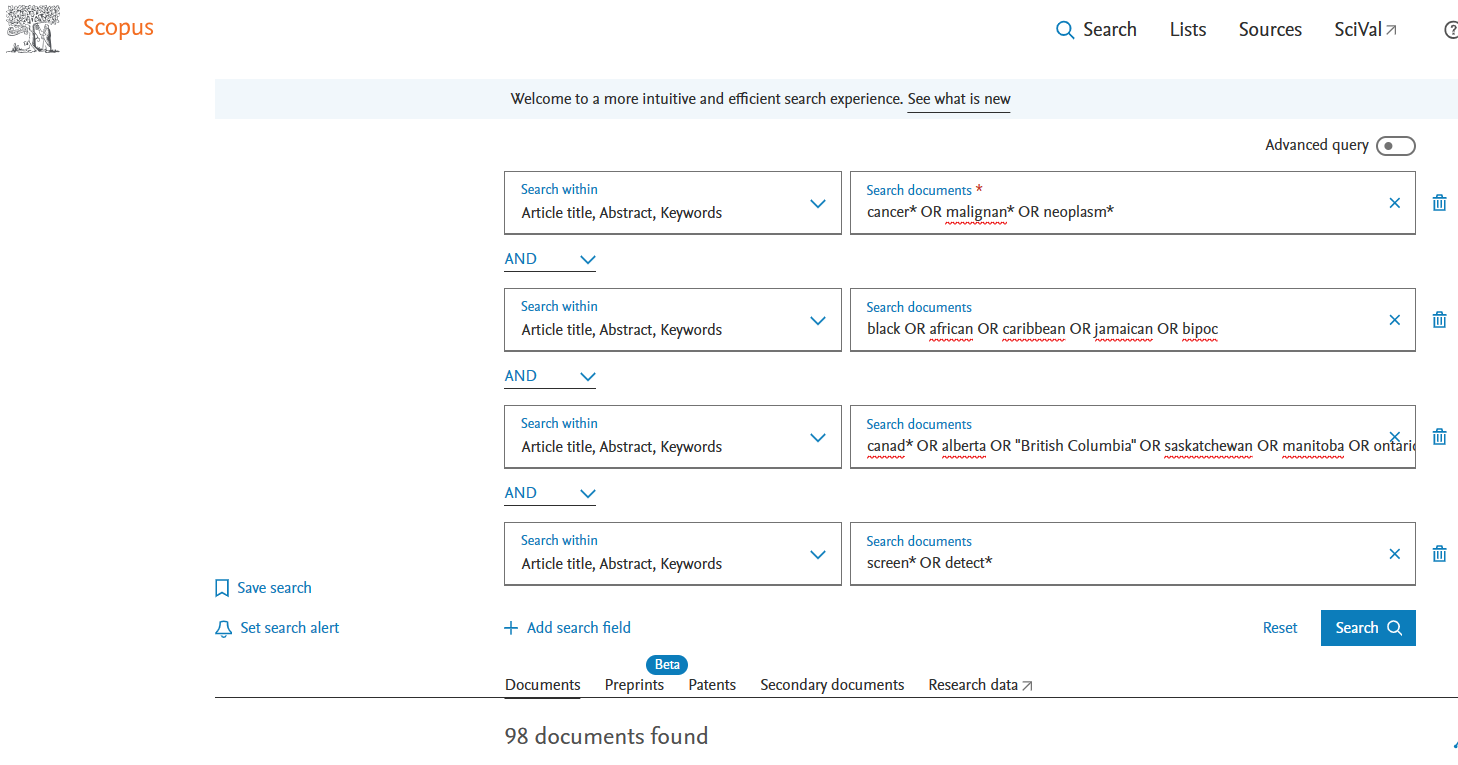


Proquest Dissertations & Theses Global 5 results

abstract(detect* OR screen* ) AND abstract(cancer* OR malignan* OR neoplasm* ) AND abstract(Black OR BIPOC OR African OR Caribbean OR Jamaican) AND abstract(Canad* OR Alberta OR British Columbia OR Saskatchewan OR Manitoba OR Ontario OR New Brunswick OR Nova Scotia OR Prince Edward Island OR Newfoundland OR Yukon OR Northwest Territories OR Nunavut)

Sociological Abstracts 2 results

summary(detect* OR screen* ) AND summary(cancer* OR malignan* OR neoplasm* ) AND summary(Black OR BIPOC OR African OR Caribbean OR Jamaican) AND summary(Canad* OR Alberta OR British Columbia OR Saskatchewan OR Manitoba OR Ontario OR New Brunswick OR Nova Scotia OR Prince Edward Island OR Newfoundland OR Yukon OR Northwest Territories OR Nunavut )

SocIndex with Full Text 4 results

detect* OR screen* [Select a Field]

**AND** cancer* OR malignan* OR neoplasm* [Select a Field]

**AND** Black OR BIPOC OR African OR Caribbean OR Jamaican[Select a Field]

**AND** Canad* OR Alberta OR British Columbia OR Saskatchewan OR Manitoba OR Ontario OR New Brunswick OR Nova Scotia OR Prince Edward Island OR Newfoundland OR Yukon OR Northwest Territories OR Nunavut [AB Abstract or Author-Supplied Abstract]


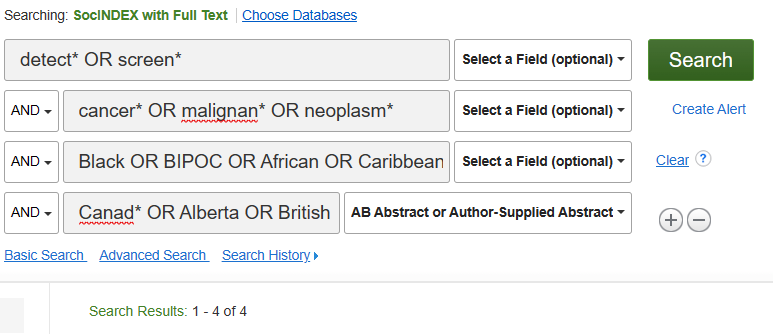

Supplement: Supplementary file 2 — Supplementary Material 2. [file 12889_2026_27317_MOESM2_ESM.docx]
